# A *k*-mer grammar analysis to uncover maize regulatory architecture

**DOI:** 10.1186/s12870-019-1693-2

**Published:** 2019-03-15

**Authors:** María Katherine Mejía-Guerra, Edward S. Buckler

**Affiliations:** 1 Institute for Genomic Diversity, Cornell University, 175 Biotechnology Building, Ithaca, 14853 NY USA; 2 USDA-ARS, Research Geneticist, USDA ARS Robert Holley Center, Ithaca, 14853 NY USA; 3 Department of Plant Breeding and Genetics, Cornell University, 159 Biotechnology Building, Ithaca, 14853 NY USA

**Keywords:** Gene regulatory regions, Machine learning models, Crops genomics

## Abstract

**Background:**

Only a small percentage of the genome sequence is involved in regulation of gene expression, but to biochemically identify this portion is expensive and laborious. In species like maize, with diverse intergenic regions and lots of repetitive elements, this is an especially challenging problem that limits the use of the data from one line to the other. While regulatory regions are rare, they do have characteristic chromatin contexts and sequence organization (the grammar) with which they can be identified.

**Results:**

We developed a computational framework to exploit this sequence arrangement. The models learn to classify regulatory regions based on sequence features - *k*-mers. To do this, we borrowed two approaches from the field of natural language processing: (1) “bag-of-words” which is commonly used for differentially weighting key words in tasks like sentiment analyses, and (2) a vector-space model using word2vec (vector-*k*-mers), that captures semantic and linguistic relationships between words. We built “bag-of-*k*-mers” and “vector-*k*-mers” models that distinguish between regulatory and non-regulatory regions with an average accuracy above 90%. Our “bag-of-*k*-mers” achieved higher overall accuracy, while the “vector-*k*-mers” models were more useful in highlighting key groups of sequences within the regulatory regions.

**Conclusions:**

These models now provide powerful tools to annotate regulatory regions in other maize lines beyond the reference, at low cost and with high accuracy.

**Electronic supplementary material:**

The online version of this article (10.1186/s12870-019-1693-2) contains supplementary material, which is available to authorized users.

## Background

The majority of sequence polymorphisms that are statistically associated with phenotypic variation (GWAS) lie in the non-genic portion of the genome, where they might play regulatory roles [1, 2]. Recently biochemical characterization of the open chromatin space in B73 (the maize reference line), revealed that as much as 40% of the significant sequence polymorphisms - as identified through variance components analyses – overlap with regions in which regulatory elements are expected [3]. These biochemical assays are prohibitively expensive and time consuming at the scale of breeding programs for any crop species. This is even more true for species, such as maize, with high genomic diversity and a high rate of polymorphism. Similar to other crops, in maize, less than half of the genome sequence is expected to be shared between inbred lines [4]. Building accurate models from expensive data derived from reference line(s) will enable breeders to project that information to other genotypes for use in genomic selection models and to prioritize regions of the genome to edit using strategies such as CRISPR technology [5, 6].

The most common models to annotate a non-coding sequence with a regulatory role is the use of collections of transcription factor binding sites (TFBSs), or “motifs”, usually in the form of Position Weight Matrices (PWMs). Collections of PWMs are usually derived from large scale experiments (in-vivo or in-vitro) capable of biochemically characterize the interactions between proteins and the DNA. In plants, only in Arabidopsis, large collections of PWMs describing TF:DNA interactions are available. Franco-Zorrilla JM et al. and O’Malley RC et al. [7, 8]. For plant regulatory regions, a number of convenient tools to identify “motifs” from sets of sequences, or to identify candidate regulatory regions based on the presence of PWMs are routinely used in molecular biology relying on Arabidopsis annotations across species [9, 10]. As a shortcoming “motifs” are elusive, it is common to have experimental data from TF:DNA interactions from which a PWM can not be obtained [11]. When available, PWMs are limited in their application to identify candidate regulatory regions, frequently achieving poor recognition performance [12, 13].

Most of the experimental and computational approaches used to annotate functional non-coding regions focus on the regulatory role of TFBSs [14, 15]. However, it has been observed that patterns of sequence organization (the grammar) and the chromatin context in which TFBSs are located contribute to the regulatory message [16–18]. For instance, the spatial arrangement of poly(dA:dT) tracts within yeast promoter regions have been identified as causal drivers of transcriptional patterns at comparable levels to TFBSs [19]. More recently, it was shown that developmental enhancers in *Ciona* rely on the positioning, arrangement, and space between TFBSs to counterbalance low TFBS affinity [20]. From this emerging view, it appears that regulatory regions have distinctive features that can be exploited for prediction, identifying enriched key sequences and sequence organization.

The frequency of oligomers of length *k*(i.e., short *k*-mers in the size range of TFBS) have been exploited to build supervised models capable of discriminating regulatory regions from random genomic regions, as well as to score sequence variation with few or no assumptions regarding to the role that a given *k*-mers might play [21–23]. The early *k*-mers count-based classifiers have been improved to count gapped *k*-mers, allowing exploration of short and long *k* values without losing power as the total number of *k*-mers increases [24]. Some limitations of *k*-mers frequency-based methods include: (1) they make poor or no use of the *k*-mers positional relationships in their models, and (2) they perform poorly in the presence of repetitive regions, the frequencies of short size *k*-mers are misleading, which might hamper the performance of this methods for genomes with high repeat content.

Recently however, a growing set of computational tools using Neural Networks (NNs) have shown success in learning to recognize simple sequence patterns, similar to PWMs. These approaches have been able to further integrate those patterns into more complex features to discriminate regulatory regions [25–27]. Generally, the NNs implemented for genomic data are Convolutional Neural Networks (CNNs), a type of architecture that shows state-of-the-art performance for key phrase recognition tasks in Natural Language Processing (NLP), but not Recurrent Neural Networks (RNNs) which are preferred for comprehension of whole sentence semantics given their power in modeling long-span relations [28, 29]. Despite their power, CNNs are often implemented in a black-box context and interpretation of their output is challenging; thus it remains unclear how much of their performance is derived from recognizing key motifs, motif relationships, and the general sequence context. For these reasons we choose to implement *k*-mer approaches rather than CNN’s or RNN’s.

To define sequence arrangements with putative regulatory roles, we analyzed the architecture of regulatory regions at the *k*-mer level, focusing on weighted individual frequencies and co-occurrences, while considering a genome environment with high repeat content. The core of the analysis builds on machine learning approaches commonly applied in the natural language processing (NLP) community. These methods are easily interpretable and rely on word statistics to recover semantic and syntactic cues [30–33]. We evaluated the accuracy and precision of these approaches with a diverse set of functional genomics experiments to provide a comprehensive description of the regulatory landscape of the maize genome. The software implementation that allows to select control regions, train and test models, is open source and available in a public Bitbucket repository.

## Results

### Weighted frequencies and co-occurrences of short sequences can accurately discriminate regulatory from random genomic regions

To build accurate classifiers we collected a comprehensive set of regions enriched in regulatory function (hereafter, ’regulatory regions’), as identified in B73 (maize reference genome) through different biochemical assays. We included in the open chromatin regions by MNA-seq derived from two tissues [3], binding loci from ChIP-seq peaks of two TFs (i.e., Homeobox KNOTTED 1 – KN1, bZIP FASCIATED EAR4 – FEA4) [34, 35], and core promoter regions around TSSs [36–38] (Additional file 1: Table S1). Because the specific background signals from each individual experiment are not available, regulatory regions were paired with randomly chosen regions controlling for G+C content and genomic distribution. Each group of sequence (regulatory regions and their control) was separated into training and holdout sets for model evaluation. In total we analyzed 52,292,705 base pairs of regulatory regions corresponding to ∼2.5% of the effective genome size of the B73 genome.

**Fig. 1:**
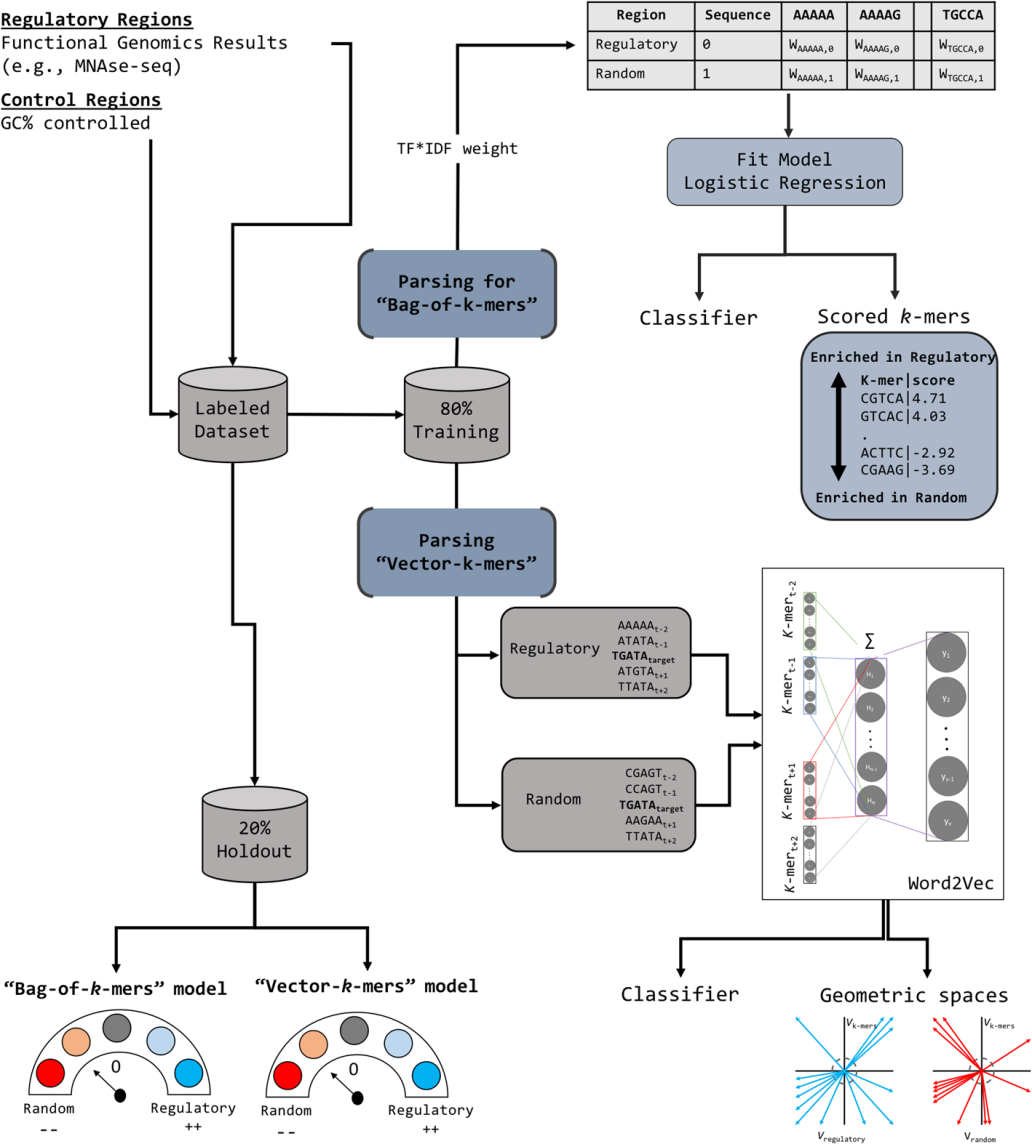
Schematic of the steps to generate “bag-of-*k*-mers” and “vector-*k*-mers” models. The workflow shows the steps from data preprocessing to model output. We fitted “bag-of-*k*-mers” and “vector-*k*-mers” models for *k* values between 5 to 10 bp (within the common range in which regulatory elements have been observed). Training and evaluation of both methods happened on the same portion of the data to facilitate comparisons. The common pre-processing step involved the collapsing of complementary *k*-mers as the same token to reduce the noise of *k*-mer counts and the effective vocabulary for feature selection. The final outputs are both the classifiers and learned features

The first part of the analysis involved the training of “bag-of-*k*-mers” and “vector-*k*-mers” models (Fig. 1). The “bag-of-*k*-mers” captures information from the *k*-mer individual frequencies and fits a logistic regression to a matrix filled with the TF*IDF (i.e., the term frequency–inverse document frequency) transformation of the raw counts per sequence [30]. Thus, the *β* coefficients of the logistic regression can be interpreted as weights of the contribution of each *k*-mer to the classifier decision and of its enrichment in regulatory and random regions. By contrast, the “vector-*k*-mers” captures information from the *k*-mer co-occurrences by training a shallow NN that learns the probability for each *k*-mer given its context (window = 5). The output is n-dimensional vectors *v*_*k*-mer_ – one per *k*-mer - independently generated for regulatory regions and their respective control (*V*_*regulatory*_ and *V*_*random*_) to denote different geometric spaces containing *v*_*k*-mer_. Next, *V*_*regulatory*_ and *V*_*random*_ are utilized to determine the likelihood of groups of *k*-mers being observed in regulatory or control regions [32, 33]. Put together, these two models aim to learn the importance of key sequence features and sequence feature relationships as descriptors of regulatory architecture.

We choose to compare our models against a “motif” collection approach. For this we used the MEME-ChIP pipeline [10]. In brief, MEME-ChIP combines several of the most popular algorithms of the MEME suite to generate PWMs (*de novo*) in a discriminative mode using the sequences in the training set. MEME-ChIP also scan sequences against a motif database from Arabidopsis [8]. The goal of this analysis was to obtain PWMs capable to differentiate between regulatory regions and control to contrast against the models. We obtained five collections, one for each type of regulatory region, of PWMs, and used it to scan the corresponding holdout sets.

**Fig. 2:**
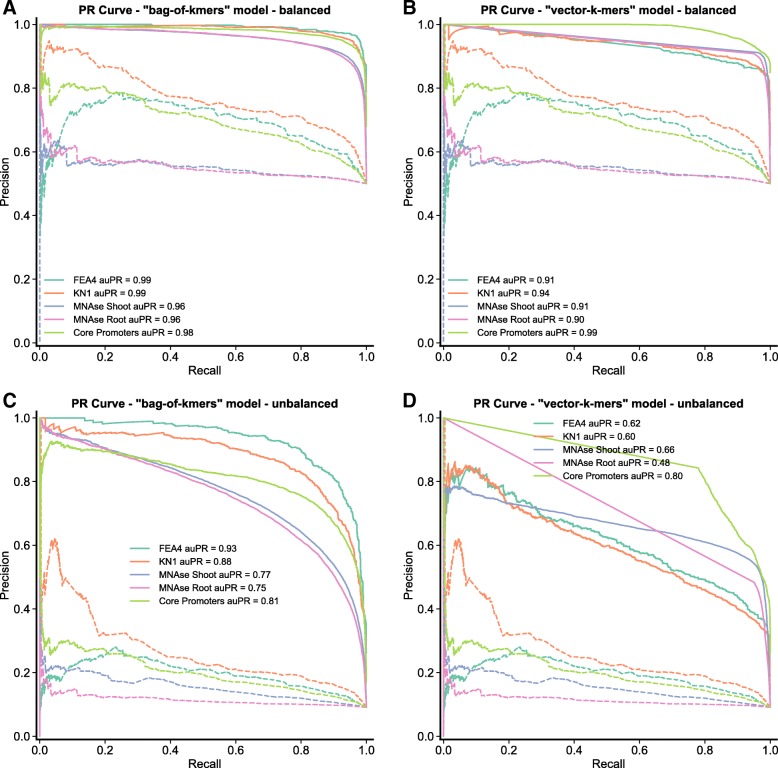
Comparison between models of the precision-recall curves. Comparison of models performance under balanced (**a** - **b**) and unbalanced holdout sets (**c** - **d**). For each model (*k*=8), the precision recall (PR) curve for all the regulatory datasets are shown, and the corresponding curves for classification of the same holdout set with a collection of PWMs (dotted lines). The PR curve shows the trade-off between precision and recall for different decision threshold. A high area under the curve represents both high recall (low false negative rate) and high precision (low false positive rate)

Model performance was measured with several metrics: (1) accuracy, precision, and recall (See “Methods” section and Additional file 2: Table S2), in addition (2) the receiver operating characteristic curve, and the precision recall curve were plotted and (3) the area under each curve was computed (auROC, auPRC) (Fig. 2a-b and Additional file 3: Figures S1). First, models were evaluated on balanced holdout sets (i.e., the same number of regulatory and random sequences). The two models perform similarly well, with average accuracy ∼90% and an average difference in accuracy of ∼3% between the two models. Overall, the “bag-of-*k*-mers” model shows better performance for most of the cases, with the “vector-k-mers” models slightly outperforming when *k* is small (*k*=5 and *k*=6) and training datasets are large (e.g., MNA-seq - shoot, root) (Additional file 2: Table S2). The collection of PWMs as an alternative classifier underperformed against all the models, in all the combinations of *k*-size and regulatory regions. Overall, PWMs appear to work better for the identification of TFBSs from TF ChIP-seq data, and for core promoter, than for the open chromatin regions (MNA-seq data) (Additional file 2: Table S2), which is expected given that enrichment of a single or few motifs is usually the landmark of TFs The performance of the “bag-of-*k*-mers” models was reliable even at *k* ≥8, as opposed to similar approaches that rely on raw *k*-mer counts as features to train machine learning classifiers [22, 39]. The above suggests that the TF*IDF transformation is efficient in alleviating some of the noise inherent to the matrix sparsity that increased with *k*.

To increase the stringency of our evaluation criteria, we measured each models’ performance with unbalanced holdout sets in which regulatory regions are outnumbered by random regions by 1 to 10 (Fig. 2c-d and Additional file 3: Figures S1C-D). Scaling up the number of random regions did not appreciably change accuracy and auROC values, but the auPRC showed a drop in model performance as the rate of false positive increased. At *k*=8, both models have a desirable precision, ∼80-70%, recovering ∼60% of the relevant regions (i.e., recall rate) for open chromatin and core promoter datasets. The “bag-of-*k*-mers” model works better for prediction of TF binding loci than the “vector-*k*-mers”, with the last one displaying an excess of false positives at our aimed recall rate (Additional file 3: Figures S2). Across a more stringent test, the PWM collections under-performed against all the other models at any given *k*, as a consequence of an increasing in the number of false positives. The performance measurement under an unbalanced set suggests that applying extra stringency to the predicted probability, thereby allowing the recovery of ∼60% of the relevant sequences, would result in an acceptable trade-off between sensitivity and specificity for most of the models when non-regulatory regions are in large numbers.

**Fig. 3:**
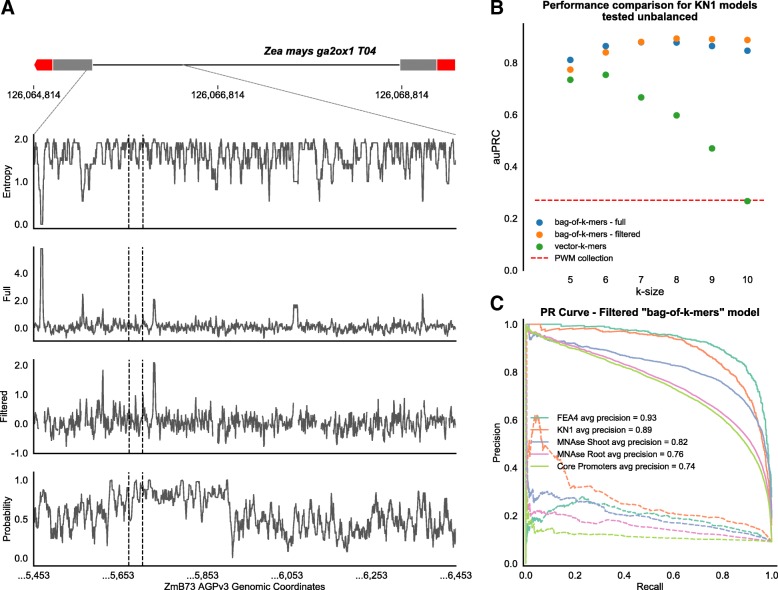
Low complexity regions do not provide relevant information to discriminate regulatory regions. **a** Annotation at a base pair level of the first 1000 bases pairs of the long intron in the maize gene *ga2ox1* using sequence complexity (Entropy), scores from “bag-of-*k*-mers” models (Full and Filtered), and regulatory probabilities (Probability) from the “vector-*k*-mers” model. Sequence complexity and “bag-of-*k*-mers” scores were calculated using a 1bp sliding window of size *k*. Regulatory probabilities were calculated using a 1bp sliding window of 3*k to evaluate co-occurrence of groups of 3 *k*-mers. The evaluated region includes the KN1 ChIP-seq peaks as identified from two biological replicates in developing ears (the center of the peak for each replicate is indicated with a vertical dotted line). **b** For all the models tested with KN1 unbalanced holdout set the performance measured as area under the PR curve shows the best performance for the “bag-of-k-mers” models at *k*=8. **c** The PR curve for (*k*=8) “bag-of-*k*-mers” filtering low-complexity k-mers shows similar performance than full 8-mer vocabulary across all the regulatory regions for the different decision threshold

Highly repetitive genomes include an abundance of low-complexity regions. These repetitive regions are expected to carry little information for regulation, and because of their high-frequency, they represent an obstacle to identifying the key elements from raw *k*-mer counts. To empirically determine a complexity threshold for *k*-mers unlikely to have a regulatory role, we examined a collection of regulatory motifs and calculated complexity (as measured with Shannon entropy) for the consensus sequences (Additional file 3: Figure S3). Using this threshold, *k*-mers with low complexity were filtered out to build “bag-of-*k*-mers” models with a reduced vocabulary (filtered), and contrasted against models using the whole vocabulary (full). The difference between the two models at a base pair level is illustrated for the *ga2ox1* first intron recognized by KN1 [34, 40]. We observed that low complexity regions overlapped with *k*-mers that have a high score from the model trained on the full *k*-mer vocabulary (Fig. 3a). This is different from the filtered model which appears to be in agreement with the ChIP-seq data (Additional file 3: Figure S4). To evaluate the importance of these repetitive sequences in recognizing the regulatory regions, we compared the models with and without low complexity *k*-mers using an unbalanced holdout set and found that both models show almost identical performance for the auROC and non-significant differences for the auPRC (Fig. 3b-c, Additional file 3: Figure S5). This suggests that in general, low complexity *k*-mers in maize do not contribute substantially to the regulatory message. However, for scaling across the genome, controlling for repetitive sequences would be critical for prediction performance and for the extraction of key k-mers that are not frequency-biased.

### Models to predict regulatory regions are scalable to the genome-wide space

Under the assumption that annotation of non-coding regions would be part of general pipelines, in which ∼85% of the genome should be recognized as repeats and ∼5% as coding sequences, our models for annotating regulatory regions should be limited to ∼10% of the space. Still, it is a challenge to accurately predict a regulatory region using a model that was training in artificial balanced data from a context that might harbor similar sequence composition while surrounded by repetitive elements. To gain insights on the behavior of the models at a genome-wide scale, the sequence of chromosome 10 was partitioned into 1,943,698 regions (300 base pairs length) and 115,149 regions that were neither repeats nor coding sequences were selected to be annotated. We used models derived from MNA-seq shoot data applying different levels of stringency for the predicted probabilities (Additional file 4: Table S3). According to the results obtained with unbalanced holdout set, and in order to balance sensitivity and specificity, we determined that the ideal predicted probability cut-off was the one that captures ∼60% of the regions that overlap with the annotated regulatory regions. Under this criteria the “bag-of-*k*-mers” (*k*=8, filtered, probability ≥0.85) and the “vector-*k*-mers” models (probability ≥0.95), predicted 38,945 and 41,932 regulatory regions respectively. The high confidence regions classified as regulatory correspond to ∼2.2–2.3% of the total regions from chromosome 10, in line with the expected portion of the genome with a regulatory function.

Next we aimed to annotate the genomes of ZmW22, a maize inbred line, that was recently made public [41]. To do so, we choose to annotate the ZmW22 genome using the MNA-seq shoot models, as open chromatin regions are usually a collection of all the regulatory regions in the genome, including promoters and TFBSs. To get a set of “ground truths” to evaluate our results we aligned ZmB73 MNA-seq regions to the ZmW22 genome, and scored windows around the alignment hits with our models. This test allow us to determine how frequently the models were able to recognize a “candidate regulatory region” in their local context, without masking the genome. This analysis evaluated regulatory vs non-regulatory regions to a ratio of 3:20, more than twice than previous presented analysis for the unbalanced holodut set

According to the observations made in the chromosome 10 of ZmB73, we used first the “bag-of-k-mers” (filtered, probability ≥0.85) to obtain the “candidate regulatory regions”. And used on top the “vector-k-mers” to obtain distances of similarities between the candidate regulatory regions and the ZmB73 MNA-seq regions summarizing region with their vector centroid distance. The combined top prediction around each of the “ground truths” resulted in an intersection with the alignment hit in a ∼70% of the cases. Allowing up to three top predictions around each hit, increases to ∼77% of the cases.

### Models trained in maize can be used to inspect the regulatory space in related species

**Fig. 4:**
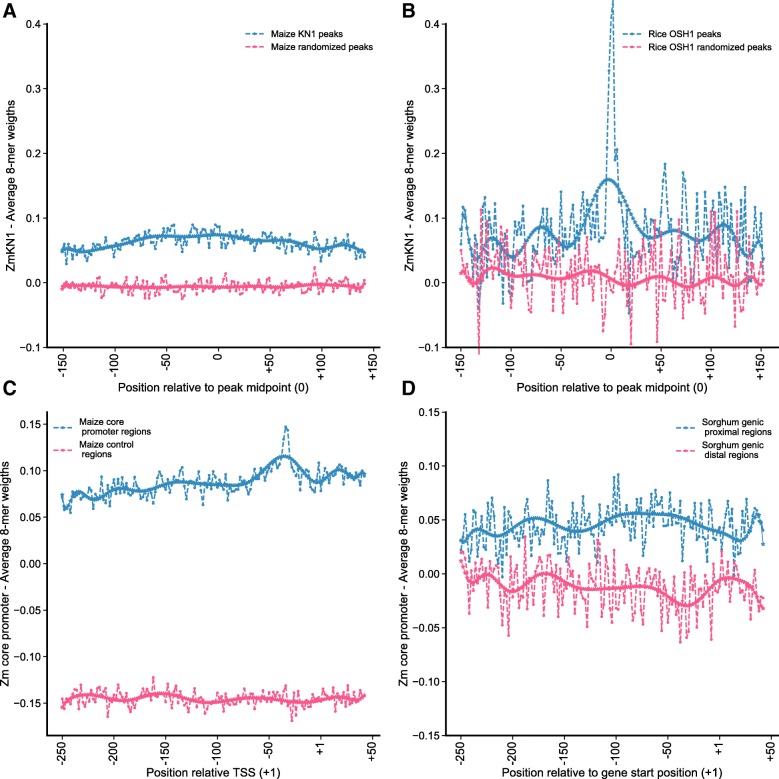
Prediction of Core Promoter Regions and TF Binding Loci Across Species. **a***k*-mer weights derived from a “bag-of-*k*-mers” model trained in maize (Zm) KN1 regions were used for the base-pair annotation of KN1 binding loci (blue) and control regions (red). **b** The same weights were used to annotated regions from rice, and to differentiate regions targeted by OSH1 (the functional orthologue of KN1) [42] (blue) from control regions (red). **c** A “bag-of-*k*-mers” model trained in maize core promoters (Zm) and tested in maize. **d** Model trained in maize core promoters was tested in in sorghum (Sb) for a set of randomly chosen 1000 core promoter regions and their respective controls

Transference of functional genomic annotations across diverse maize lines requires models than can preferentially capture conserved features (those common between lines or related species). Consistently, we expect that models that are accurate in related species should also perform well in different maize lines. To gain insights into this we evaluated models trained on TF binding loci and core promoters in two species (sorghum and rice). In order to determine positional preferences among binding loci, we built peak meta-profiles that summarized KN1 models’ performance in maize and rice at the base-pair level (Fig. 4a-b). The “bag-of-*k*-mers” model can differentiate between regulatory regions and their control in maize, and in addition can distinguish rice KN1-like (i.e., OSH1) binding sites (i.e., peaks from rice OSH1 ChIP-seq data [42]). On the other hand, the “vector-*k*-mers” cannot differentiate between random regions and regulatory regions in rice, predicting random as regulatory (Additional file 3: Figure S6A). Interestingly, the distributions of regulatory probabilities for random and regulatory regions are noticeable different (Additional file 3: Figure S6B), suggesting that the “vector-*k*-mers” model distinguish between OSH1 peaks and control regions, but not enough to assign greater non-regulatory probability to random regions. In maize, the “bag-of-*k*-mers” model (filtered) shows an slight preference towards the midpoint region versus the edges, while the “vector-*k*-mers” recognizes the whole region without preference for to the middle (Fig. 4a). In rice, the “bag-of-*k*-mers” shows a marked preference near or at the peak midpoint over the flanking (Fig. 4b). This suggests that the “bag-of-*k*-mers” capture a diverse array of features which are enriched at the center of the peak and beyond in maize. However, only the key features that are enriched at the center of the peak appear indeed conserved between the two species.

For the evaluation of models trained on core promoters we used a balanced holdout set derived from a random sample of sorghum annotated gene models. The positional preferences in core promoters in maize are evident from average *k*-mers weights around the +30 region, in which a TATA-box is expected (Fig. 4c). The same is not observed in Sorghum (Fig. 4d). This likely result from the biased sample of TSS in maize that have a high proportion of TATA+ promoters, even when TATA-less promoter are the majority [38]. A positional analysis using the “vector-*k*-mers” models did not reveal local enrichment along the sorghum promoter sequences. Yet, the probabilities scores are again different between control sequences and core promoter sequences. The difficulties of the model to identify control regions might be a consequence of the strong differences between the repeat landscape in the non-coding regions between sorghum and maize that is not captured in the maize training set, rather than a lack of similarities between the regulatory regions of the two species. Taken together we have shown that classifiers trained in maize can be useful to predict regulatory regions in sorghum and rice, and that features enriched in maize regulatory regions and in the random genomic space (as captured by the models) are of two general types: (1) maize specific and (2) conserved across related species.

### Scored vocabularies highlight signatures of regulatory function

The methods proposed here were chosen because of the interpretability of the learned features, aiming to better understand the patterns in sequence that characterize regulatory regions. Thus, we focused on scored *k*-mer vocabularies (*k*=8, filtered) as easiest to interpret, and systematically analyzed the tails of the distribution as they concentrated the most informative sequences. Therefore, the largest positive coefficient values (top scored *k*-mers) are indicative of enrichment and the largest negative values (bottom scored *k*-mers) of depletion in regulatory regions. The absolute values from both sides of the score distribution are different, with preference for positive over negative ones, meaning that model’s prediction are the result of identifying those *k*-mers that are enriched in regulatory regions rather than depleted ones (or enriched in random regions). We found that properties of the scored *k*-mers obtained from applying an out-of-the-box NLP technique [32] are similar to those previously described with sequence kernels developed to analyze vertebrate genomic data [22–24].

**Fig. 5:**
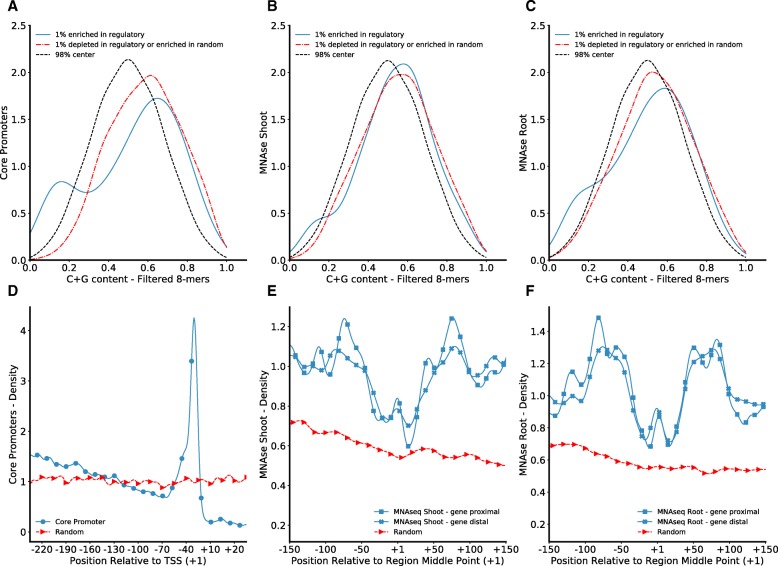
G+C Content Bias is Related to Positional Constrains within Promoters and Open chromatin Regions. Comparison of the distribution of G+C content across top 1%, bottom 1% and remaining 98% of scored *k*-mer vocabularies (*k*=8, filtered) for **a** core promoters, MNA-seq **b** shoot and **c** root model’s results. The positional constraints of *k*-mers with high A+T content on the top 1% visualized as k-mer’s density with respect to a reference point: **d** TSS for core promoters and MNA-seq hotspot middle point for open chromatin regions **e** shoot and **f** root (solid blue lines). Contrasting density plots are shown for corresponding random regions (dotted gray lines)

We observed a bias in the G+C content at the extremes of the score distribution for core promoters (Fig. 5a) and to a lesser extend for open chromatin regions (Fig. 5b-c). The 1% of the top shows a bimodal distribution, in which a subpopulation of *k*-mers exhibits low G+C content, in contrast to the 1% of the bottom, and the remaining 98%. Conversely, the score distribution for TF binding loci shows a general shift of top and bottom tails towards higher G+C contents, in comparison to the remaining 98% (Additional file 3: Figure S7). These results are in agreement with known roles for high A+T sequences within core promoters related to the TATA elements and high G+C sequences as TF binding sites [38, 43]. Indeed, when investigated, individual *k*-mers with high A+T content were positionally restricted upstream of the TSS and preferentially on the region defined for the TATA element in maize (Fig. 5d).

The enrichment of MNA-seq regions for *k*-mers with high A+T content (rich A+T *k*-mers) might be derived from signal co-localization between open chromatin regions and core promoters [3]. If signal co-localization were sufficient to explain the similarities between open chromatin and core promoter regions, then controlling for distance to annotated genes should remove the signal from rich A+T *k*-mers in distal regions. Yet, controlling for near gene proximal (2kb) the positional constraints remain in both, proximal and distal, regions (Fig. 5e-f). These rich A+T *k*-mers might be part of poly(dA:dT) tracts which can provide an increase in DNA rigidity and are known to be in proximity to regions that are enriched in TFBSs [44]. In agreement with the positional restriction, rich A+T *k*-mers flank the midpoints where G+C content is high, as expected for the regions that are bound by TFs [43], and where the signal for open chromatin regions is concentrated.

In addition to key structural tracts, *k*-mers with the largest positive values for each regulatory category are expected to be enriched for TF motifs. Because the number of experimentally verified maize motifs is limited, we contrasted the top 1% of positive scored *k*-mers against two large collections of TF motifs as identified from large scale experiments in the reference plant *Arabidopsis thaliana* (TOMTOM, p-value <0.001) [7, 8] (Additional file 5: Table S4). For the evaluated experiments we found that the top 1% of positive *k*-mers are ∼threefold more enriched for significant hits against the motif database than expected by chance for all the *k*-mers in the population. The enrichment for the top *k*-mers was statistically significant (hyper-geometric test, p-value <0.001). Further analyses revealed that *k*-mer scoring is consistent within families of TF binding sites. In particular, motifs preferentially hit by the top 1% of positive *k*-mers from FEA4 binding loci (a bZIP transcription factor) correspond to the bZIP/TGA-class, and motifs preferentially hit by *k*-mers enriched in KN1 (a Homeobox transcription factor) correspond to the Homeobox family (Additional file 5: Table S4). Thus, the scored vocabularies produced a comprehensive catalog of *k*-mers with putative structural roles and a collection of *k*-mers similar to TFBSs that constitute signatures of the maize regulatory architecture.

### Sequence similarity in the geometric space reveals a prevalent distinctive *k*-mer organization within regulatory regions

**Fig. 6:**
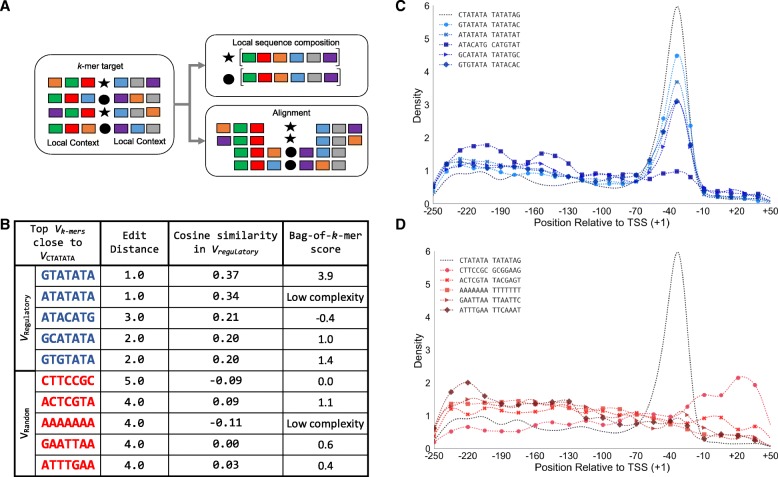
Local Sequence Context Defines Distinctive Groups of*k*-mers Between Regulatory and Random Regions. **a** Schematic of aligning flanking contexts versus contrasting local sequence composition, as implemented in the “vector-*k*-mer” models in which *k*-mers that share a similar context would be represented by close vectors (*v*_*k*-mers_) in a geometric space. **b** The vector space obtained from core promoters (*V*_*regulatory*_) and their corresponding control (*V*_*random*_) define two different groups of closest *k*-mers (cosine similarity) to the ’CTATATA’ vector (*v*_*CTATATA*_). The group of closest *k*-mers in *V*_*regulatory*_, when compared to the group formed in *V*_*random*_, are more similar in sequence (shorter edit distance), and have in average more positive *k*-mer scores from the equivalent “bag-of-*k*-mers” model. This implies a semantic-like relationship between those *k*-mers in regulatory sequences versus random regions. **c** The group of *k*-mers closest in the *V*_*regulatory*_ space have similar positional preferences (blue solid lines) to CTATATA (black dotted line) in the region expected for the TATA element. **d** In addition, the group of *k*-mers closest in the *V*_*random*_ (red solid lines) do not show similar positional constraints to CTATATA (black dotted line) do not show positional preferences relative to the TSSs

The set of highly enriched individually scored sequences, as output from “bag-of-*k*-mers” models, is likely to include groups of *k*-mers that correspond to the same motif, given the degeneracy of TFs binding sites. However, the question arises of how to group *k*-mers that likely share functional roles and constitute single motifs. In NLP, word2vec is an effective method to extract linguistic regularities between words by considering the local context in which they occurs (e.g., apple and oranges might share local contexts as they are words with similar meanings) [45]. Because vector position in each geometric space is determined from the composition of the local word/*k*-mer context (i.e., neighboring *k*-mers), we can assume that two k-mers that are close (i.e., close in cosine distance) to each other in a geometric space share local sequence similarity (Fig. 6a). Therefore, we used the geometric spaces obtained from the “vector-*k*-mers” models, to extract *k*-mer regularities or *k*-mer organizational ’rules’ that differentially arise between regulatory and random regions. Because, the position of *k*-mers between geometric spaces cannot be directly contrasted, we compared the lists of closest *k*-mers for any given *k*-mer in the vocabulary as obtained from the geometric spaces about regulatory and random regions (respectively, *V*_*regulatory*_ and *V*_*random*_).

To illustrate, we compared the representative vector of the *7*-mer *CTATATA* in *V*_*regulatory*_ (i.e., set of *v*_*k*-mers_ learned from core promoter regions) and in *V*_*random*_ (i.e., set of *v*_*k*-mers_ learned from random regions used as controls for core promoters). Using *v*_*CTATATA*_ we obtained the set of top five closest *v*_*k*-mers_ in *V*_*regulatory*_ and in *V*_*random*_ and found that *k*-mers from *V*_*regulatory*_ share more sequence similarity (average edit distance 1.8 vs 4.2 respectively) and have, on average, more positive scores from the respective “bag-of-*k*-mers” model (1.49 vs 0.01) (Fig. 6b). In addition, *k*-mers close to *v*_*CTATATA*_ in *V*_*regulatory*_ share positional constraints that are not recovered from those related in *V*_*random*_ (Fig. 6c-d). This example shows how the output of the geometric spaces can be exploited to determine groups of similar *k*-mers according to their context.

To obtain a global view of how many *k*-mers are embedded in different local sequences between regulatory and random regions, we collected for any given *k*-mer (*k*=8) in the vocabulary, the list of the closest similar *k*-mers ranked by cosine similarity from *V*_*regulatory*_ and *V*_*random*_. Next, we contrasted the two ranked lists and determined which *k*-mers show the greatest dissimilarity between regulatory and random regions [46]. In general, we found that low complexity *k*-mers do not show distinctive organizational ’rules’ between regulatory regions and random, reinforcing our view that short repetitive sequences are not important to define the identity of a sequence. We found that, in terms of the number of *k*-mers with different relationships between *V*_*regulatory*_ and *V*_*random*_, “vector-*k*-mers” models derived from TF binding loci (∼45%) and core promoter regions (∼30%) result in notably more differentially represented *k*-mers than models derived from open chromatin regions (∼5%). In all the cases, we observed a similar proportion of *k*-mers enriched and depleted in regulatory regions (as established from the “bag-of-*k*-mers” scores). The results from models trained in open chromatin regions, might represent the heterogeneity of the regions that prevents the model from learning many specific *k*-mer vectors. However, the fact that the classifiers work with great accuracy indicates that even when the differences are less pronounced than for TF binding loci and core promoter regions, they are large enough to distinguish between an open chromatin region and its control.

We integrated the information obtained from the “bag-of-*k*-mers” and the “vector-*k*-mers” models and found that for the top 1% of the *k*-mers that are enriched in frequency in regulatory regions there is little overlap between *k*-mers that resemble motifs and *k*-mers that show differential relationships between regulatory regions and random regions. For instance, from the FEA4 models, only 10 out of 103 *k*-mers, that are statistically similar to Arabidopsis motifs, show differential *k*-mer relationships between regulatory and random regions. Such difference might be derived from the proportion of TFBSs that are not similar between Maize and Arabidopsis *cis*-regulatory elements. In summary, we have compiled a regulatory vocabulary that includes a proportion of key k-mers that are enriched in regulatory regions and (1) resemble known motifs, and (2) are embedded in a specific regulatory context.

## Discussion

The decreased cost of large scale genotyping and genome assemblies for crops such as maize and related species, has already shown potential to accelerate the breeding process by linking sequence and structural variation to phenotype [47]. A majority of functional genetic variation that is important to phenotype is located in the non-coding regions of the genome. This variation is largely untapped because recognizing functional alleles in the non-coding regions of the genome is both expensive and laborious. In humans and other metazoan models, non-coding annotation that allows identification of functional genetic variation has been accelerated over the last decade using two types of analyses: (1) functional analysis from large collections of biochemical assays; and (2) comparative sequence analysis between reference genomes of closely related species [48]. Yet, in maize, these two types of analyses are particularly challenging. Large collections of biochemical assays remain prohibitive at the scale necessary to cover maize diversity, which is 20 times more than the diversity found in humans [49]. In addition, comparative sequence analysis requires genome alignment between closely related species, which for maize and its relatives is complicated by the presence of a large number of repetitive sequences in the genome.

In this study, we introduce a computational framework consisting of two type of machine learning models that can accurately classify regulatory regions obtained from functional genomic experiments and random genomic regions. These approaches were borrowed from the fields of natural language processing and information retrieval, and were explicitly chosen to overcome the challenges of annotating intergenic regions in maize. To address highly repetitive sequences and the role of low-complexity regions in maize non-coding regions the “bag-of-*k*-mers” model relies on first filtering out *k*-mers with low-complexity, and next using a sub-linear function to transform raw *k*-mer frequencies to down weight *k*-mers that are too frequently observed in a group of sequences and in consequence have less power to discriminate between regulatory and non-regulatory regions. In parallel, the “vector-*k*-mers” model learns local *k*-mer organization from *k*-mer co-occurrence frequencies, which in practice results in a geometric space that allows alignment-free comparisons between sequences [50]. The simultaneous use of two different approaches adds robustness to the predicted annotations, allowing researchers to contrast or to combine the results of the two types of models.

In most of the functional genomics experiments the expectation is to identify rare instances of a biochemical event (e.g., the locations in the genome in which the chromatin is accessible for enzymatic digestion) versus thousands of instances that represent noise. Learning from imbalanced data occurs frequently in many machine learning applications. However, in machine learning rare instances (in our case regulatory regions) are treated as noise. So, training with the true genomic ratio of regulatory:non-regulatory regions will cause the models to learn non-regulatory features over regulatory ones. In the maize genome, non-regulatory features will be the ones that characterize the most abundant class of repeats. On the other hand training in re-sampled data (balancing the ratio of regulatory:non-regulatory region), generate models that expect a distribution of instances that strongly differs from the genomic distribution of events. We decided to pose the problem in a way that the models could learn features from regulatory regions. Next we used a series of evaluations with “real-world” constraints to adjust the probability cut-offs at which the models predictions are still reliable while taking care of the excess of false positives. We show that the adjustment of the probabilities *a posteriori* and the combined use of the two models allow us to “transfer” annotations from ZmB73 to ZmW22 with reasonable precision.

Because both models are amenable to interpretation, examination of the learned features offers novel insights about key sequence characteristics that can help to build mechanistic hypotheses to be tested at molecular level, and allow comparison of regulatory programs under the same framework. For instance, both types of models suggest that low complexity *k*-mers are not important for regulatory regions in maize. The comparative use of the models shows that TFBSs (i.e., FEA4 and KN1) are better predicted with the bag-of-*k*-mers. Also, through modeling MNA-seq data we found that open chromatin regions in maize are characteristically organized within poly(dA:dT) tracts flanking G+C rich *k*-mers resembling motifs (Fig. 5a-b). Likewise, from modeling maize KN1 ChIP-seq data and further annotation of regions bound by OSH1, we determined conservation at the center of binding loci for the key individual *k*-mers (Fig. 4b) and a lousy conservation in the pattern of *k*-mer co-occurrences (Additional file 3: Figure S6A). These results suggests that, though the non-coding regions change rapidly across species, the use of sequence models allows alignment-free comparisons to determine regulatory features that are conserved across million years of evolution.

## Conclusions

Taken together, our framework can be used beyond the transference of regional annotations, as can easily be extended to evaluate *in silico*, the putative effect of sequence variation (i.e., SNPs, single nucleotide polymorphisms) in regulatory function from the differences in k-mer scores and regulatory probabilities for small groups of *k*-mers.

This work opens many avenues for improving models by adding relevant layers of information. Possible layers to add include: predictions of the 3D structure of regulatory regions, joint modeling of functional genomic data spanning the range of maize diversity to identify general patterns for relevant phenotypes, or even extended across species to build more generalizable models that capture the most conserved features. Furthermore, we expect these annotations to be useful as priors to improve marker assisted technologies such as genomic selection to purge deleterious non-coding sequence variation and to identify targets for genome editing contributing to gene expression dysregulation.

## Methods

### Definition of maize regulatory regions

In the analyses presented throughout this study, we used data sets derived from different functional genomic experiments and obtained from the reference genome (ZmB73 AGPv3, chromosomes 1 to 10) [51]. We included in the analysis open chromatin regions in shoot and roots derived from MNA-seq data [3]; binding loci for KNOTTED 1 (KN1) and FASCIATED EAR 4 (FEA4) transcription factors from ChIP-seq data [34, 35], and promoter regions [36–38] from the intersection of TSSs obtained with CAGE and FLcDNAs (Additional file 1: Table S1). For MNA-seq hotspots, ChIP-seq, we collected sequences of 300 base pairs length symmetrically surrounding the midpoints from the originally defined regions. Similarly, for core promoters, we selected the region between -250;+50 base pairs surrounding the TSSs. Each group of regulatory regions was randomly divided between training and holdout sets and reserved for further analyses. Training and testing was performed independently for each type of regulatory regions.

To randomly select control regions, we search in the vicinity (maximum in a 100 kb window) around a given regulatory region for a control region that have a matching G+C content and does not overlap with any of the other regulatory region; if no match was found, we removed the vicinity criteria and searched for a G+C matching region in the same chromosome. For the holdout sets we build balanced and unbalanced sets from randomly selecting one, and ten control regions, respectively, for each regulatory one.

### Definition of grasses regulatory regions

Sorghum (*Sorghum bicolor*) core promoter regions were obtained from the reference genome (v2.1) [52] for the coordinates between -250;+50 base pairs surrounding the start position of genes with annotated 5’UTR and a subset of 1000 sequences randomly selected for further analyses. Rice (*Oryza sativa Nipponbare*) KNOTTED 1-like (i.e., OSH1) binding regions were obtained from re-analyzing ChIP-seq experiment starting with the download of raw data from DDBJ (http://www.ddbj.nig.ac.jp/) (accession numbers DRA000206 and DR000313) corresponding to two biological replicates of immunoprecipitation with *α*-OSH1 and IgG antibodies [42]. Raw reads were mapped against the rice reference genome (IRGSP-1.0 [52]), using bowtie v1.1.2 (options -n 2, -l 60, -X 500, –best, –strata, -m 1) [53] and low quality and duplicated reads were removed using picard (http://broadinstitute.github.io/picard/) (MarkDuplicates) and samtools (options -F 780, -F 1024, -f 2) [54] MACS v2.1.0 [55] was used for peak calling (options -g 3.73e8, -q 0.01) for each of the replicates and 42 peaks with a reproducible absolute summit reserved and further extended to 300 base pairs for downstream analyses. Corresponding control regions were obtained as explained above for maize. Briefly, each reference genome was divided into windows and after removal of sequences overlapping the putative regulatory regions we randomly selected sequences matching G+C content and when possible in the vicinity (∼10 kb) of each of the regulatory sequences.

### Preprocessing of sequences

Sequences were preprocessed before fitting models. The preprocessing for the “bag-of-*k*-mers” model involves the dividing of each sequence into 1 base pair sliding (overlapping) windows of a given size *k* (*k*-mers) to collect for a sequence of length L (L-*k*)+1 *k*-mers. Next, *k*-mers were converted into tokens (*t*) that correspond to collapsed pairs of *k*-mer and their respective reversed complementary. For the “vector-*k*-mers” models, each sequence is described as a collection of “sentences” resulting from walking *k* times and sliding by 1 base pair. Each sentence is broken into ordered non-overlapping new tokens. For testing sentences are divided in neighborhoods to obtain regulatory and non-regulatory likelihoods for groups of *k*-mers

### Calculation of TF*IDF and implementation of the “bag-of-*k*-mers” model

Let’s define all the sequences in a given set from a functional genomics experiment and its corresponding control regions as a collection *S*={*s*_1_,*s*_2_,…*s*_*n*_} of individual sequences. Next, for each individual sequence *s*_*i*_ let’s define a set of tokens *T*_*i*_={*t*_1_,*t*_2_,…,*t*_*n*_}. All the possible tokens for a given *k* belong to the vocabulary, *Y*. Each *T*_*i*_ is mapped to a list of token weights - *W*_*s*_- of size | *Y* | that contains “weights” for each token that occurs in *T*_*i*_, where the “weight” (Eq. 1) is defined as the product of the token frequency - *f(t)* - in *s*, and its inverse collection frequency - *idf(t)*-. Calculation of TF*IDF were done according to the implementation in the python library scikit-learn v0.19.0 [56].

1 $$  weights(s,t) = f(t) log \frac{1 + |S| }{|s \in S : t \in T| + 1}  $$

To generate a “bag-of-*k*-mers” model, each training data set is represented as a *x* matrix, with Ws -list of token weights- as rows, and a list *y* of sequence labels (1 for regulatory regions and 0 for control regions). The “bag-of-*k*-mers” model results from fitting a regression curve, *y = f(x)* (i.e., a logistic regression). The C parameter for the logistic regression was chosen by fivefold cross-validation using a grid search function. Logistic regression and grid search functions as used here correspond to the implementation of the python library scikit-learn v0.19.0 [56].

### Implementation of “vector-*k*-mers” model

To generate “vector-*k*-mers” models we used the implementation of word2vec algorithms from the python library gensim v1.0.0, which fits sequence representations (*k*-mer vectors - *v*_*k*-mers_) via Stochastic Gradient Descent (SGD) that aims to optimize an objective function, that implicitly correspond to likelihood for *k*-mer co-occurrences [32, 57]. Next, as shown for text classification, sequence representations -*v*_*k*-mers_- can be turned through inversion via Bayes rule to determine the likelihood of a new sequence of being part of a regulatory region based on its *k*-mer composition [33]. This classification schema interprets the individual *v*_*k*-mers_ as components in a composite likelihood approximation that allows classification of sequences without extra modeling or estimation steps.

In brief, we trained a shallow (one single hidden layer), fully connected neural network aimed to optimize the probability of predicting a given *k*-mer (*k*-mer_target_) from its context, that is from the observation of the co-occurring *k*-mers appearing anywhere within a small window around the target. We ran word2vec with 30 iterations using hierarchical softmax and no negative sampling for each data set (options iter=30, hs=1, negative=0, size=300, min_count=0 and window=5, all others parameters were kept as the defaults) to obtain two independent geometric spaces (a continuous space of sequence representations), one for the regulatory regions (*V*_*regulatory*_) and the other for the control regions (*V*_*random*_).

For the classification step, we calculated the probability of every new sequence *s*_*i*_ under each sequence representation – *V*_*regulatory*_ and *V*_*random*_ – by first calculating the likelihood of every window within a sentence (using the score function from gensim) and the averaging likelihoods to obtain sentence likelihoods. Next, from the matrix of sentence likelihoods by the two categories (i.e., *C*= regulatory and control) we derive the sequence probabilities - *pV*_*regulatory*_(*s*_*i*_) and *pV*_*random*_(*s*_*i*_). The category probabilities were calculated via Bayes rule, using as prior *π*_*c*_=*1/C*, such that the classification proceeds by assigning the category for which *pV*_*category*_ (*s*_*i*_) is greater [33].

### Generation of PWMs collections

For any given regulatory region we generated a collection of PWMs using the MEME-ChIP pipeline, in discriminative mode. The PWMs were generated from the same training sets described above. The collection of PWMs were further used to predict on the respective holdout set. To do so, we run FIMO and consider a prediction as “positive” for any sequence with a *p*-value of less than 1e-4 for any of the motifs and a PWM scores greater than log2(10 000)=13.28 bits. This parameters have been defined as “gold-standard” to determine “positive PWMs hits” previously [12]. The collections of PWMs obtained with MEME-ChIP are available to the community at the Cyverse data store (http://datacommons.cyverse.org/browse/iplant/home/shared/panzea/dataFromPubs/Mejia2018BMCBiology)

### Models evaluation

Confusion matrix, and the Receiver Operating Characteristic (ROC) and precision recall (PR) curves were generated using the python library scikit-learn v0.19.0 [56] and plotted with python matplotlib v2.0.0 [58].

In brief, for each trained model we obtained a confusion matrix from predicting on the holdout data and compared predictions against the true categories to which each region belong. As mentioned for the training, evaluation of the model’s performance was made only in data from the same type of regulatory region in which we trained the models. It means, for instance, that only FEA4 data was used for training and evaluation of FEA4 models.

From the confusion matrix we obtained

- True positives (TP): Regions in which we predicted the regulatory category and truly belong to the regulatory category
- True negatives (TN): Regions in which we predicted the control category and truly belong to the control category
- False positives (FP): Regions in which we predicted the regulatory category, but truly belong to the control category. (Also known as a “Type I error”).
- False negatives (FN): Regions in which we predicted the control category, but truly belong to the regulatory category. (Also known as a “Type II error”)

To evaluate the models, we computed from the output of the confusion matrix the following metrics:

- Accuracy: (TP+TN)/total regions
- Precision: TP /(TP + FP)
- Recall: TP /(TP + FN)

In addition to the metrics derived from the confusion matrix we generated ROC and PR curves for each model. The ROC shows the true positive rate in function of the false positive rate for different decision thresholds (a point, sensitivity, specificity). In a ROC curve, the closer it is to the upper left corner (auROC = 1), the better the performance of the classifier. The PR curve shows the trade-off between precision and recall for different decision threshold. A high area under the curve represents both high recall (low false negative rate) and high precision (low false positive rate). The PR curve is preferred over ROC to measure the performance of a binary classifier under imbalanced datasets [56].

### Prediction of open chromatin regions in the ZmW22 genome

In order to evaluate model performance in the annotation of a non-reference maize genome we used the recently published W22 genome [41]. First we collected “ground truths” from aligning MNA-seq regions from B73 to W22 using MUMmer4, a system designed for genome alignments that can handle specie divergent DNA sequence alignments [59]. The hits in the W22 genome that correspond to the corresponding chromosome were considered “truths” or homologous regions. Next, we used the bag-of-*k*-mers models trained in MNAseq data to score overlapping (stride 150 bps) windows (lenght 300 bps) in a region corresponding to 4Kb centered in the hit. We used the vector-*k*-mers models to score each window based on their similarity to B73 MNAseq regions. For this we calculated the mean of the *k*-mers vectors to obtain a “centroid” that summarize each evaluated window to calculate the cosine similarity distance to the centroid vector of the B73 MNAseq regions. The best-scored window was compared against the hits from MUMmer4 and counted as intersecting if at least half of the length of the window was included in the MUMmer4 hit. A file with the coordinates and the predictions from each model as well as the MUMmer4 results are available to the community at the Cyverse data store (http://datacommons.cyverse.org/browse/iplant/home/shared/panzea/dataFromPubs/Mejia2018BMCBiology)

### Calculation of *k*-mer complexity on a TF motifs database

The sequence complexity of any *k*-mer was approximated to the Shannon entropy for the symbols succession given by (Eq. 2). Were *p*_*i*_ correspond to the probability of appearance of the *i*-th symbol in the *k*-mer.

2 $$  entropy(k-mer) = \sum p_{i} \log_{2} p_{i}  $$

To empirically establish a threshold of complexity for *k*-mers within regulatory regions we calculated the *k*-mer complexity for any given *k* and for all the consensus sequences derived from transcription factor (TF) binding models represented as Position Weight Matrices (PWMs) in the HOmo sapiens COmprehensive MOdel COllection (HOCOMOCO) v11 [60].

### Motif enrichment analyses

To identify *k*-mers similarity to transcription factor binding sites we used TOMTOM from the MEME suite [61] (http://meme-suite.org) and two collections of *Arabidopsis thaliana* TF binding motifs derived from large-scale experiments [7, 8]. The enrichment was calculated according to (Eq. 3), in which *N* correspond to the size of the *k*-mer vocabulary, *n* correspond to the 1% of the *k*-mer vocabulary taking from the top after sorted with the weights obtained from the model, *M* correspond to the number of *k*-mers with a significant hit against a TF motif and *m* to the number of *k*-mers that are in the top 1% and have a significant hit against a TF motif.

3 $$  enrichment = \frac{m/n}{M/N}  $$

The statistical significance of the enrichment was calculated using the hyper-geometric test, as implemented with the python library scipy 0.18.1 (stats.hypergeom) [62], after applying the Bonferroni correction for multiple testing hypothesis to the *α* (alpha) value required for statistical significance.

## Additional files

Additional file 1 Supplementary **Tables S1**. (XLSX 36 kb)

Additional file 2 Supplementary **Tables S2**. (XLSX 78 kb)

Additional file 3 Supplementary **Figures S1** to **S7**. (PDF 1253 kb)

Additional file 4 Supplementary **Tables S3**. (XLSX 39 kb)

Additional file 5 Supplementary **Tables S4**. (XLSX 66 kb)

## Abbreviations

auPRC: Area under the precision recall curve
auROC: Area under the receiver operating characteristic curve
CNN: Convolutional Neural Networks
NN: Neural Network
NLP: Natural Language Processing
PWM: Position Weight Matrix
RNN: Recurrent Neural Network
TF: Transcription Factor
TFBS: Transcription factor binding site
TF*IDF: Term frequency * inverse document frequency
