# Supplementary material for: A k-mer grammar analysis to uncover maize regulatory architecture
Source: BMC Plant Biol. 2019 Mar 15;19:103. doi: 10.1186/s12870-019-1693-2 (PMC6419808; doi:10.1186/s12870-019-1693-2)
Supplement: Supplementary file 3 — Supplementary Figures S1 to S7. (PDF 1253 kb) [file 12870_2019_1693_MOESM3_ESM.pdf]

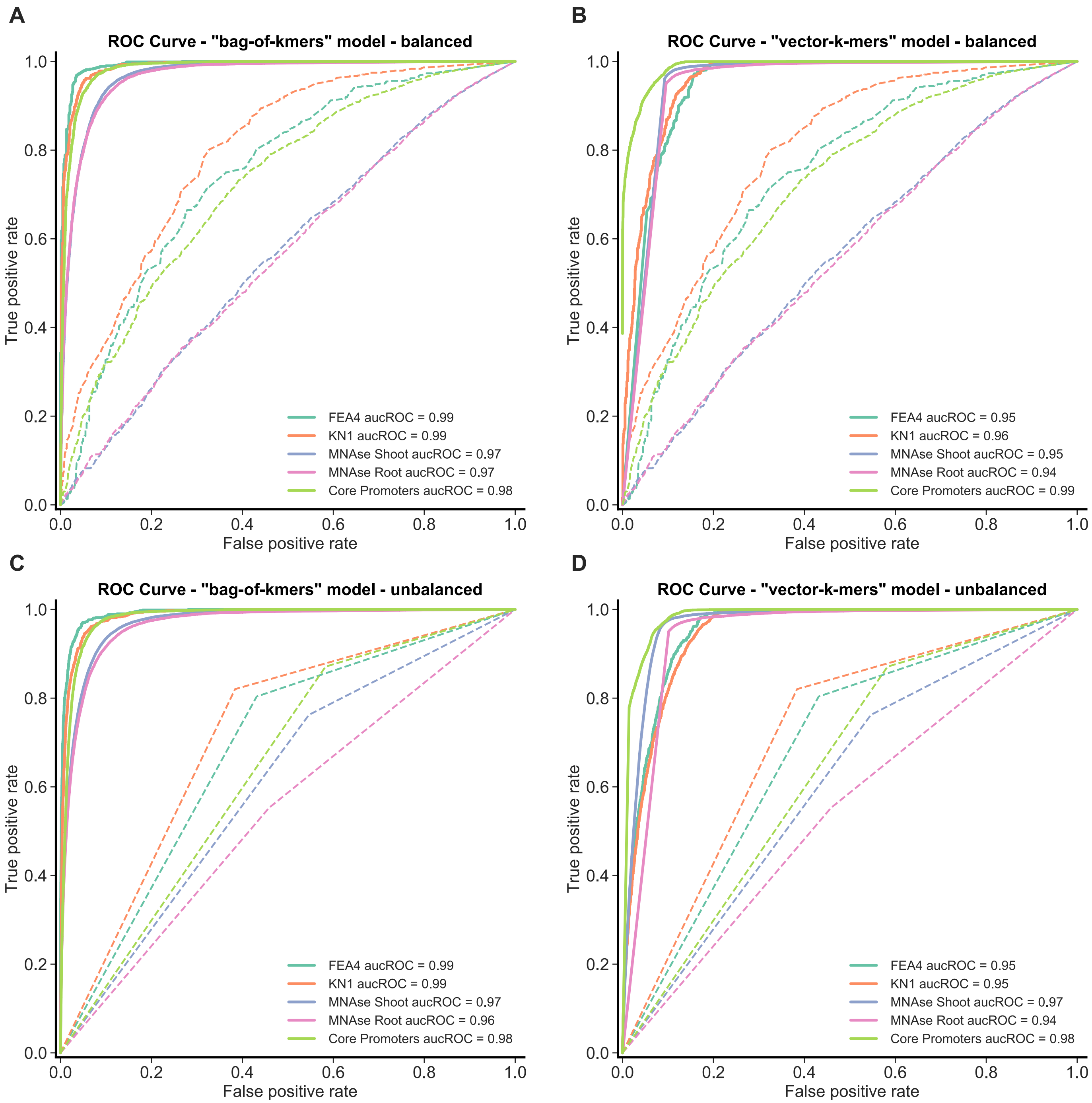

Supplemental Figure S1: Comparison between models of the receiver operating characteristic curve.

Comparison of models performance under balanced (A- B) and unbalanced holdout sets (C - D). For each model *k*=8), the receiver operating characteristic (ROC) curve for all the regulatory datasets across different machine learning models are shown (solid lines), and the corresponding curves from a collection of PWMs (dotted lines). The ROC curve illustrate the trade-off between true positives and false positives at different decision thresholds. A high area under the curve represents high sensitivity and high specificity. Curves that approximate to the diagonal are closer to the random expectation.

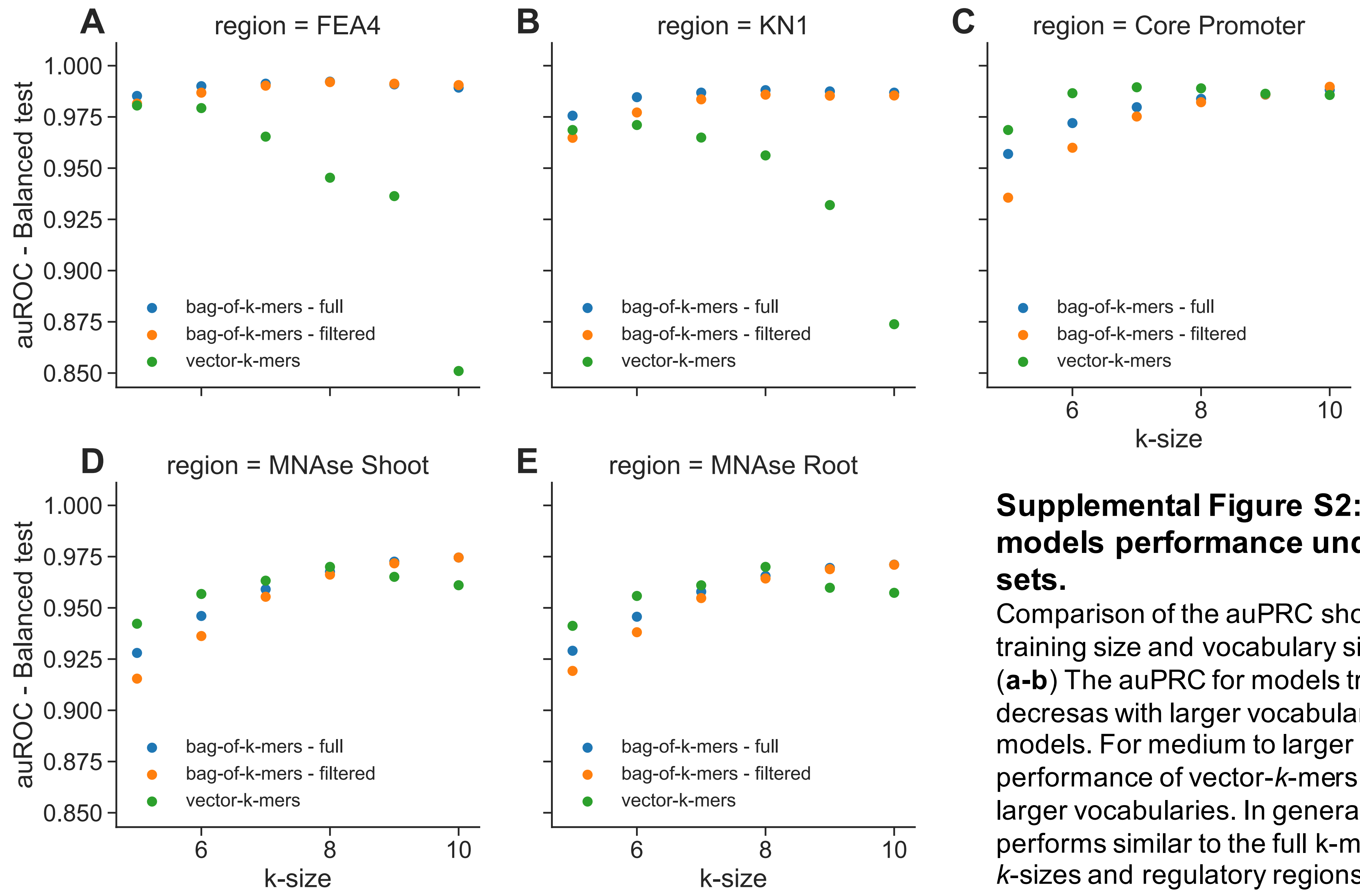

Supplemental Figure S2: Comparison of models performance under balanced holdout sets.
Comparison of the auPRC shows the effect of the training size and vocabulary size for model performance. (**a-b**) The auPRC for models trained in FEA4 and KN1 decresas with larger vocabularies for vector-*k*-mer models. For medium to larger datasets (**c-d-e**), the performance of vector-*k*-mers is still acceptable for larger vocabularies. In general the filtered k-mer models performs similar to the full k-mer models across different *k*-sizes and regulatory regions

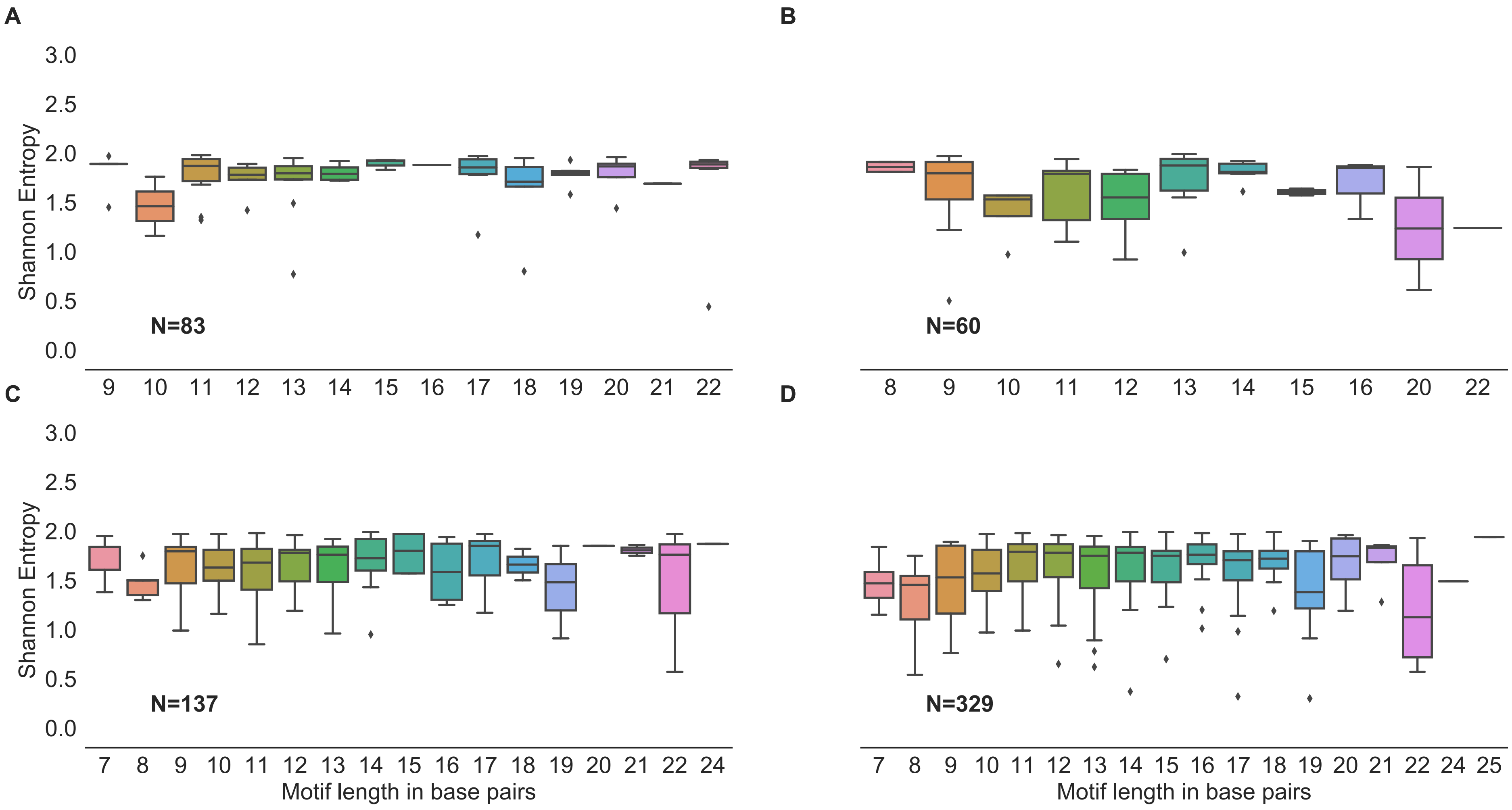

Supplemental Figure S3: Shannon Entropy's Distribution of The HOMOCOCO Motif Collection.

Distribution of the entropy for *k*-mers derived from the consensus motifs (PWMs) hosted in the HOmo sapiens COmprehensive MOdel COllection (HOCOMOCO) v11 [60]. Distributions are shown by the level of confidence of the motifs, as defined in the HOCOMOCO collection, and displayed from highest confidence motifs to lowest. High quality models, that characterize TF binding affinities are shown in (**a**), (**b**) and (**c**). Models with limited predictive power or limited evidence are shown in (**d**). Number of models for each confidence category are indicated as Ns.

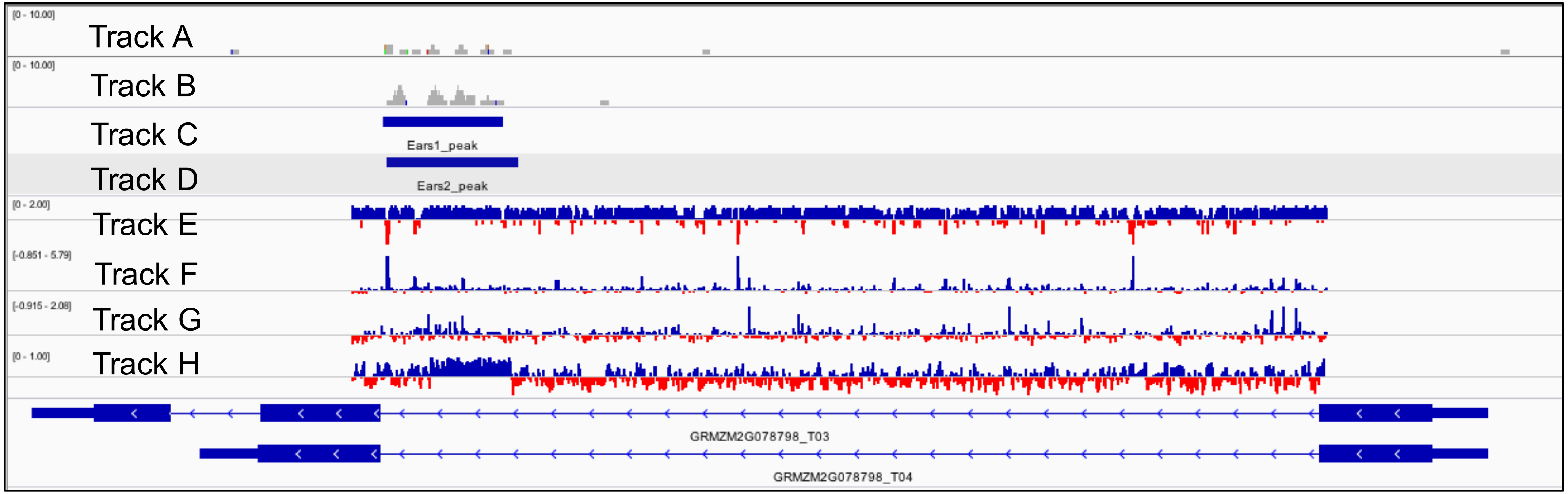

Supplemental Figure S4: Genome Viewer Screenshot of KN1 ChIP-seq alignments and KN1 Models Annotations

View in the genome browser of the region corresponding to the first intron of *ga2ox1* (gene models GRMZM2G078798_T03 and T04 in the ZmB73_AGPv3). Tracks correspond to the coverage from alignment files of two KN1 ChIP-seq biological replicates (Tracks A and B) [34], peaks position from each biological replicate as determined with MACS v2.1.0 (Tracks C and D) [55], and annotation at a base pair level using sequence complexity (Track E, Shannon entropy), scores from “bag-of-*k*-mers” models (Track F, Full and Track G, Filtered), and regulatory probabilities (Track H, Probability) from the “vector-*k*-mers” model. Sequence complexity and “bag-of-*k*-mers” scores were calculated using a 1bp sliding window of size *k*. Regulatory probabilities were calculated using a 1bp sliding window of 3*k* to evaluate co-occurrence of groups of 3 and 2 *k*-mers.

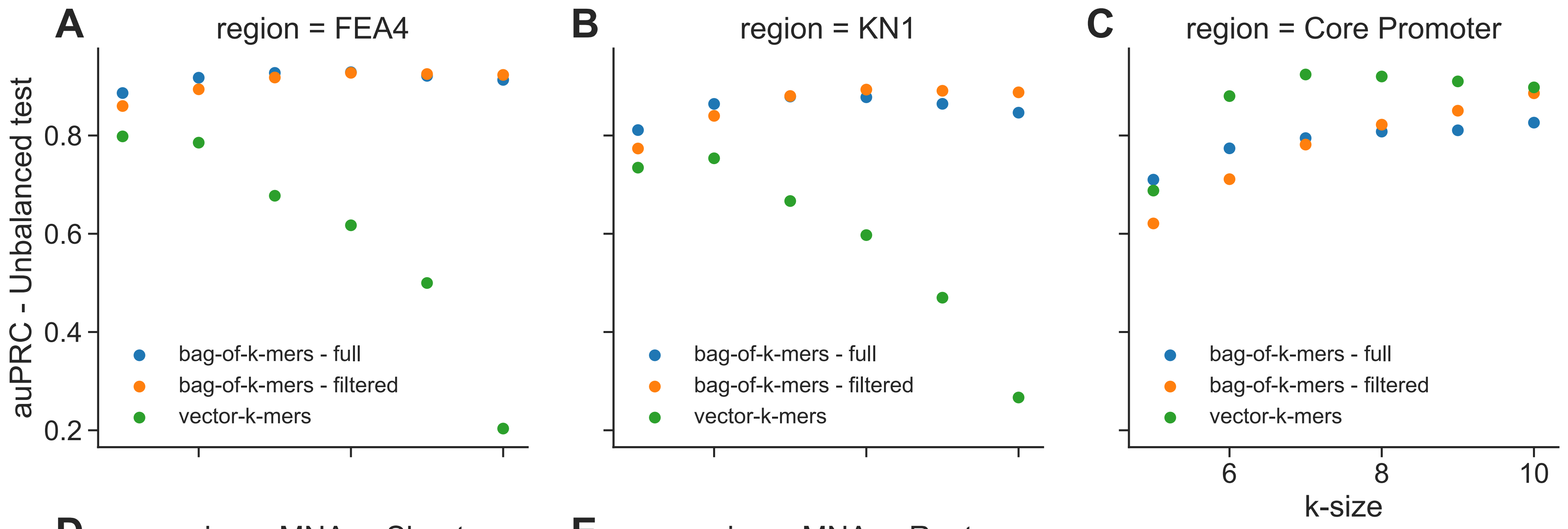

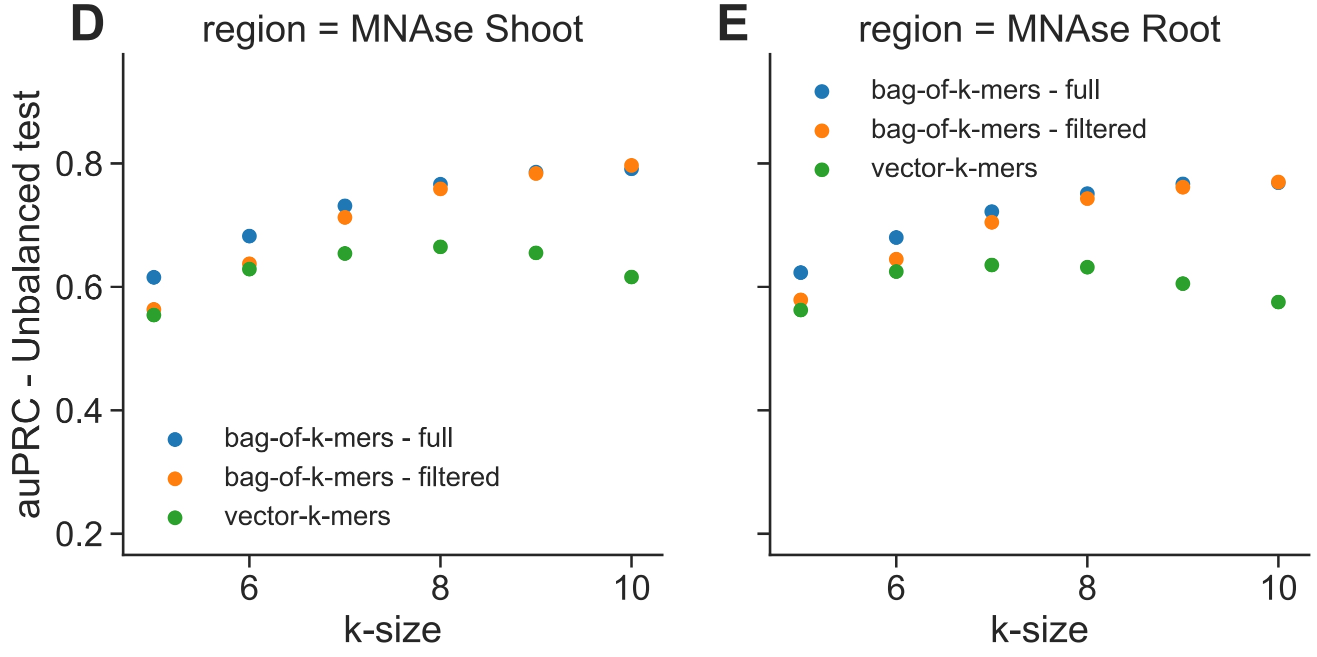

Supplemental Figure S5: Comparison of models performance under unbalanced holdout sets. Comparison of the auPRC shows the effect of the training size and vocabulary size for model performance. (**a-b**) The auPRC for models trained in FEA4 and KN1 decreases with larger vocabularies for vector-*k*-mer models. For medium to larger datasets (**c-d-e**), the performance of vector-*k*-mers is acceptable for k-size 5 to 7. In general the filtered k-mer models performs similar to the full k-mer models across different *k*-sizes and regulatory regions.

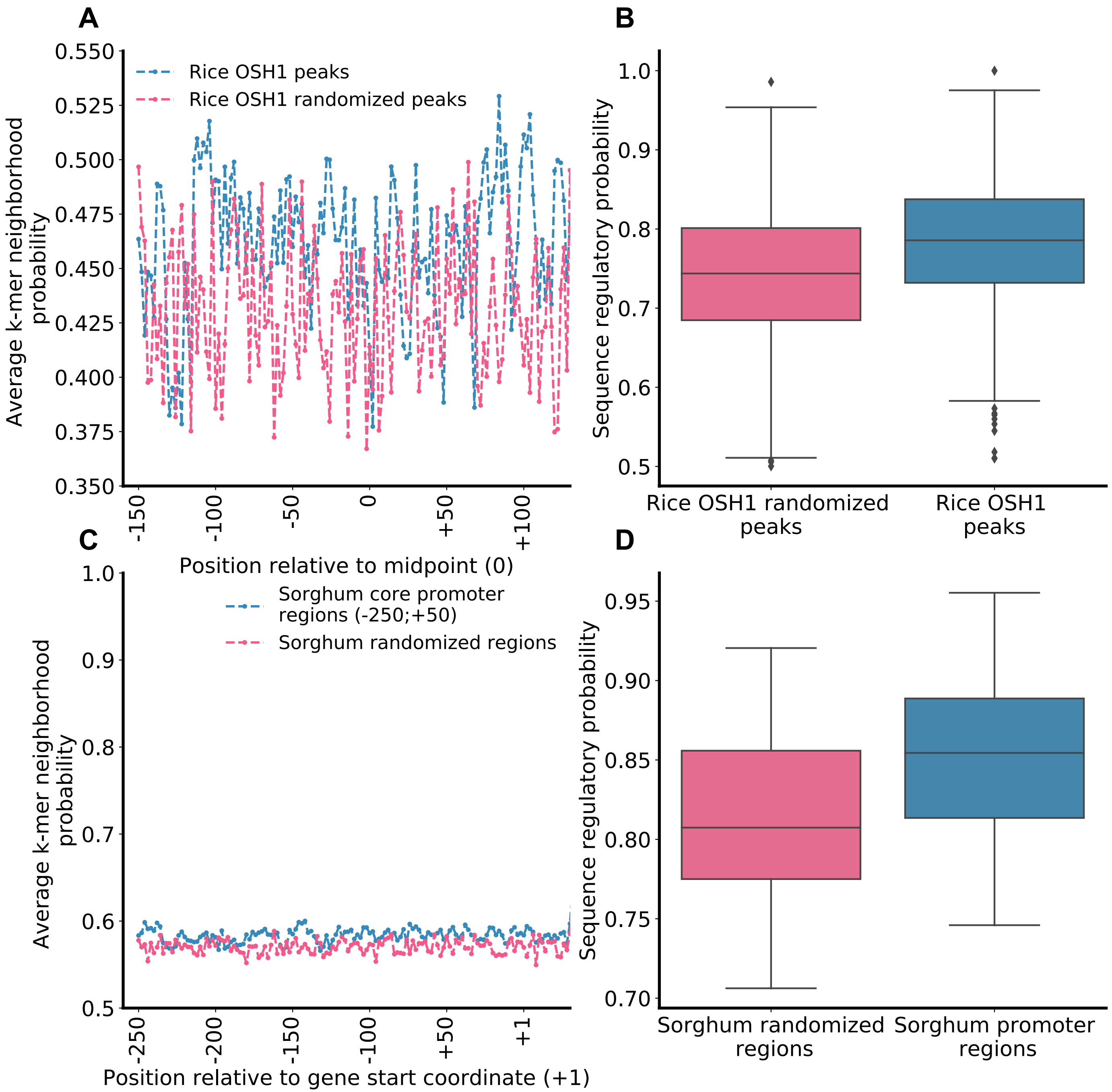

Supplemental Figure S6: "vector-*k*-mer" Maize's Model Annotations Across Related Species

(a) Local annotation of OSH1 binding loci (blue) and control regions (red) using probability scores derived from a model trained on maize KN1 (KNOTTED 1, ZmHD1) ChIP-seq data [27]. Along the sequence groups of *k*-mers are in average predicted as non-regulatory regions. (**b**) Probability scores are significantly different between rice OSH1 target regions and control regions, however for both the regulatory probability suggest regulation.
(c) Local annotation of Sorghum core promoter (blue) and control regions (red) using probability scores derived from a model trained on maize regions around experimentally determined TSSs. The model doesn't differentiate between local groups of *k*-mers . **(d)** Probability scores are significantly different between sorghum promoter regions and control regions, however for both (regulatory and control) the regulatory probability suggest regulation.

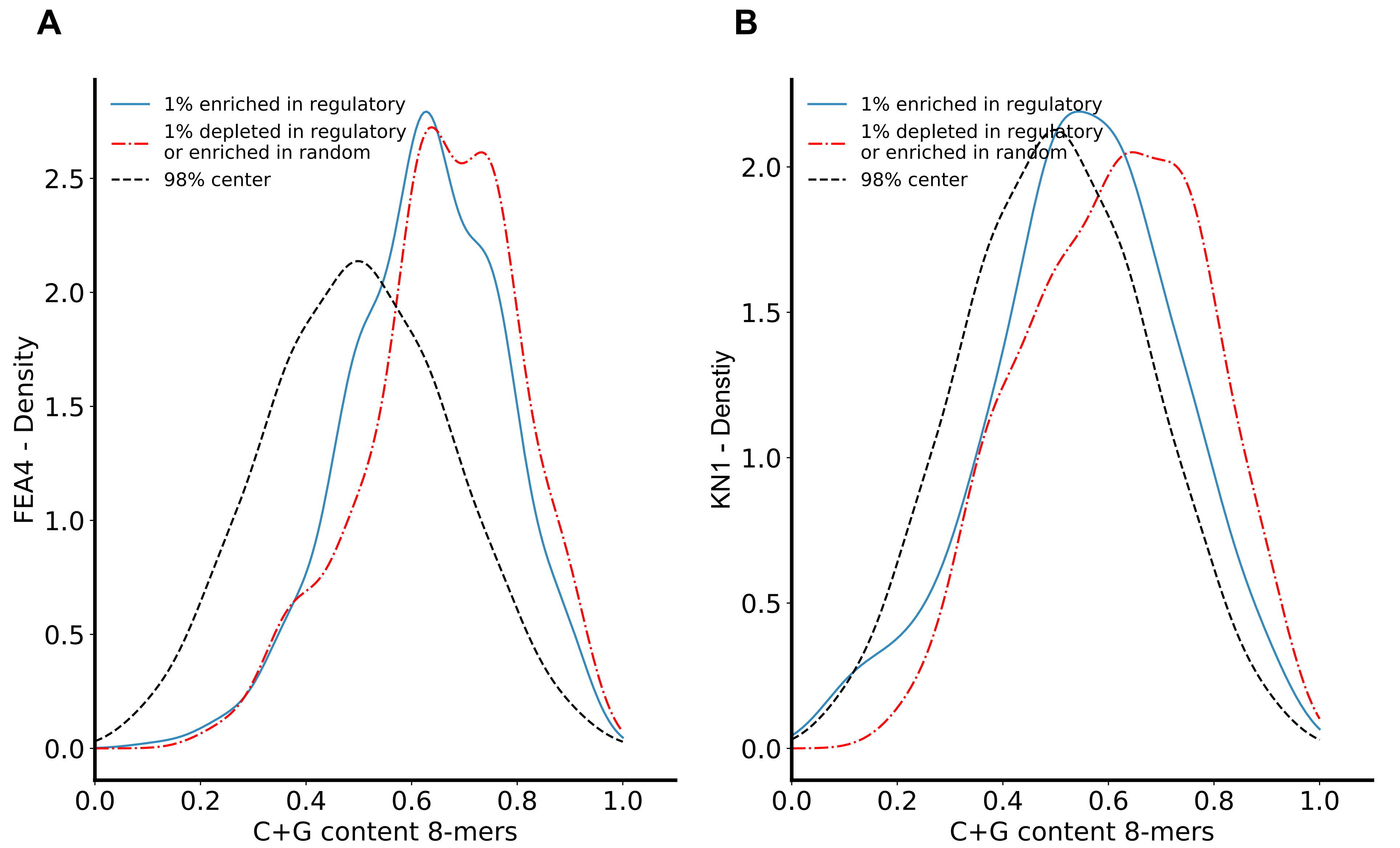

Supplemental Figure S7: KN1 and FEA4 Distribution of G+C Across the Scored *k*-mer Vocabulary

Comparison of the distribution of G+C content across top 1%, bottom 1% and remaining 98% of scored *k*-mer vocabularies (*k*=8, filtered) for models trained in the datasets (**a**) KN1 and (**b**) FEA4.
